# The Impact of the Covid-19 Pandemic on Iranian Oil and Gas Industry Planning: A Survey of Business Continuity Challenges

**DOI:** 10.1007/s13753-022-00412-7

**Published:** 2022-05-03

**Authors:** Seyyed Abdollah Razavi, Ali Asgary, Marjan Khaleghi

**Affiliations:** 1 Department of Energy Economics and Management, Tehran Faculty of Petroleum, Petroleum University of Technology, 1453953153 Tehran, Iran; 2 Disaster & Emergency Management, School of Administrative Studies, Faculty of Liberal Arts & Professional Studies, York University, Toronto, ON M3J 1P3 Canada; 3 Department of Industrial Engineering, Abadan Faculty of Petroleum, Petroleum University of Technology, 6318714317 Abadan, Khuzestan Iran

**Keywords:** Business continuity, Covid-19 pandemic, Global disaster, Iran Oil and gas industry, Pars Oil and Gas Company

## Abstract

The Covid-19 pandemic has severely affected various aspects of life, and its compounding and cascading impacts have been observed in most industries and firms. The oil and gas (O&G) industry was among the first to experience the impacts as the pandemic began due to the global economic recession and a sharp decline in demand for oil. The pandemic revealed major risk management and business continuity challenges and uncovered some of the vulnerabilities of the O&G industry and its major companies during a prolonged global disaster. Examining and understanding how the Covid-19 pandemic impacted the O&G sector in different countries, considering their unique circumstances, can provide important lessons for managing the current and future similar events. This study investigated various impacts of the Covid-19 pandemic on the O&G industry using Iran’s Pars Oil and Gas Company (POGC) as a case study. Data were collected through in-depth interviews with key managers of the company. Qualitative methods, specifically thematic analysis, were used to analyze the data. Findings of this study provide further insights into how the pandemic impacted the operations, risks, and business continuity of the POCG. The results show that the pandemic caused significant operational, financial, and legal impacts by disrupting routine maintenance, reducing the availability of human resources under the public health measures and mobility restrictions, increasing processing and delivery times, increasing costs and decreasing revenues, and delaying contractual obligations.

## Introduction

The oil and gas (O&G) industry experienced a downturn as the Covid-19 pandemic started, owing largely to the global economic recession and the decline in demand for oil across the world. This decline was the result in particular of public health interventions that aimed to reduce physical contacts by restricting mobility, and of the closure of nonessential socioeconomic activities. The O&G industry is a significant sector in the economy of oil-producing countries by employing a large number of people and by producing a significant portion of the gross domestic product (GDP). The O&G industry has been hit hard while trying to make sure that it can continue to operate during the pandemic and meet essential demands. As such the O&G industry has been a crucial part of the fight against the impacts of the pandemic by supplying energy without interruption, while complying with the public health measures to protect its own employees. Although the O&G industry is not unfamiliar with disruptive and disaster events caused by natural, technological, and human-made hazards, the Covid-19 pandemic has been substantially beyond the industry’s past experiences. Past pandemic events such as the 2003 SARS, 2006 H5N1, and 2009 H1N1 outbreaks had limited and sometimes mixed (negative/positive) spatiotemporal impacts at national and global levels (Asgary et al. 2020; Qin et al. 2020). However, during Covid-19 the situation has been very different because the pandemic negatively impacted the O&G industry in many countries at the same time. The Covid-19 pandemic revealed major challenges and issues that the O&G industry and its major companies can face during a large-scale and prolonged global disaster event. Most large O&G companies activated their business continuity and pandemic plans and followed public health measures to minimize staff shortages and other operational and financial impacts. Despite these plans, the Covid-19 pandemic uncovered some of the vulnerabilities of this sector and its major players and created complex business continuity management challenges for which most companies were not adequately prepared.

Understanding how the pandemic impacted the O&G sector and different companies in different countries considering their unique circumstances is very important. Such understanding can create pathways for implementing measures to mitigate such impacts in the future (Dube et al. 2021). This study aimed to investigate the impacts of the Covid-19 pandemic on the O&G industry using Iran’s Pars Oil and Gas Company (POGC) as a case study. We particularly examined various operational, financial, human, contractual, and safety impacts and challenges that the Covid-19 pandemic created for the POGC. Section 2 provides a brief review of the current research in this area. Section 3 describes the research methods, including the case study and data collection approaches, and Sect. 4 presents the main findings followed by a brief discussion and conclusions in Sects. 5 and 6.

## Literature Review

Studies of the impacts of Covid-19 and how different businesses, particularly those in the O&G industry, have responded to and managed the crisis are still very limited. The impacts and management approaches used by the industry and firms during the Covid-19 pandemic can be examined from the supply, demand, and operational sides. The supply side focuses on the impacts of the pandemic on the supply of materials, resources, and information to the O&G industry and its firms. The demand side focuses on the impacts of the pandemic on the demand for goods and services produced by the industry and its firms. The operational side concerns the impacts of the pandemic on the daily operations of the firms in the industry.

Demand for O&G products decreased during the pandemic (Norouzi et al. 2020; Jiang et al. 2021; Shupler et al. 2021). Travel restrictions, border closures, and closure of nonessential activities and stay and work from home all decreased demand for all modes of transport, which slowed down demand for fuel by transportation, manufacturing, and non-essential businesses and activities (Mofijur et al. 2021). Lower production and transportation reduced the demand for and consumption of liquid fuel, and the long-term storage of crude and refined O&G.

Reduction in demand for O&G can create major operational issues for O&G companies through corrosion failure problems that can adversely affect the efficiency, storage, and transportation of the petroleum refineries (Verma et al. 2021). The reduction in demand was also caused by indirect impacts of the pandemic on incomes and further reduction in fuel consumption by households and businesses (Barbosa et al. 2020). But combating the Covid-19 pandemic also created some new demands for the O&G industry and firms, particularly those serving the health sector (Barbosa et al. 2020). Operationally, most businesses activated their pandemic response and business continuity plans, if they had one, to provide a safe workplace for their employees and continue their operations. Possible reductions and absentees in the workforce concerned many companies at the beginning of the pandemic. Therefore, to protect the health and well-being of employees and their working conditions, companies implemented various risk mitigation measures as guided by public health agencies and industry best practices. Attention to these aspects can help with enhancing employee morale and productivity and can save companies considerable direct and indirect costs (Maamri 2021). Despite these measures, the Covid-19 pandemic negatively impacted corporate performance (Huayu et al. 2020), particularly in firms operating in the O&G sector, due to the interconnectivity between different processes. During the pandemic, O&G companies were not able to cover fixed costs and expenses, which resulted in poor performance. Low performance often led to low corporate revenues (Maamri 2021).

So far, few studies have examined the Covid-19 pandemic impacts on the O&G industry as a whole. Ponkratov et al. (2020) studied Russian oil industry development, considering the specifics of the current economic environment given the Covid-19 pandemic. They employed multifactor correlation modeling in combination with the three-sigma rule and fuzzy sets to predict demand and supply for Russian oil for 2021−2022. They concluded that the level of industry revenues and growth rates will be significantly lower than the pre-pandemic level, and this can continue for a long period of time. In response to the challenges brought by the Covid-19 pandemic, the O&G industry accelerated digital transformation. Hawash et al. (2020) studied the impacts of the Covid-19 pandemic on the O&G sector digital transformation. They showed that the pandemic forced companies to seek innovative and quick solutions to continue their activities and improve operational efficiencies. By adopting digital technologies, O&G companies were able to reduce the short-term economic consequences of the pandemic. They argue that digital transformation needs to be considered as a long-term continuity and resilience solution, and companies need to create a strategic vision and consider financial support for effective digital transformation (Hawash et al. 2020).

A limited number of studies are available on the impacts of the Covid-19 pandemic on O&G companies. Kee et al. (2021) studied the impacts of the pandemic on the Petronas O&G company in Malaysia. They demonstrated that the pandemic impacted Petronas revenues mostly through the falling oil price. They also described how Petronas responded to the crisis and managed business continuity by taking strategic measures, such as continuous employee salary payments, work from home, maintaining fiscal discipline, and strict health and safety measures to minimize the outbreak and risk of Covid-19 spreading in the company (Kee et al. 2021). In another recent report, Adrian (2021) examined the Covid-19 impacts on British Petroleum (BP), in particular how BP responded and managed the Covid-19 crisis considering its 2010 Gulf of Mexico oil spill crisis. Using data and information collected through interviews with BP employees, they found that BP had plans in place to manage the demand and operational impacts of the pandemic. They argue that BP has had numerous experiences in dealing with price fluctuations and this was not an exception. They also show that working remotely was not a big challenge for BP, because the company had strong remote working capabilities (Adrian 2021). Like many other businesses, the O&G companies have been successful in their ability to implement work from home as a business continuity measure. This shows the importance of more agile and cost-effective practices. Companies have learned to adapt to emerging situations very quickly (Dewi and Adiarsi 2020). At the same time, many O&G companies reduced their employees or salaries during the Covid-19 pandemic to manage their budget (Menhat et al. 2021). This research contributes to the body of knowledge by uncovering the impacts of Covid-19 on the POGC in Iran.

## Research Method

**Fig. 1:**
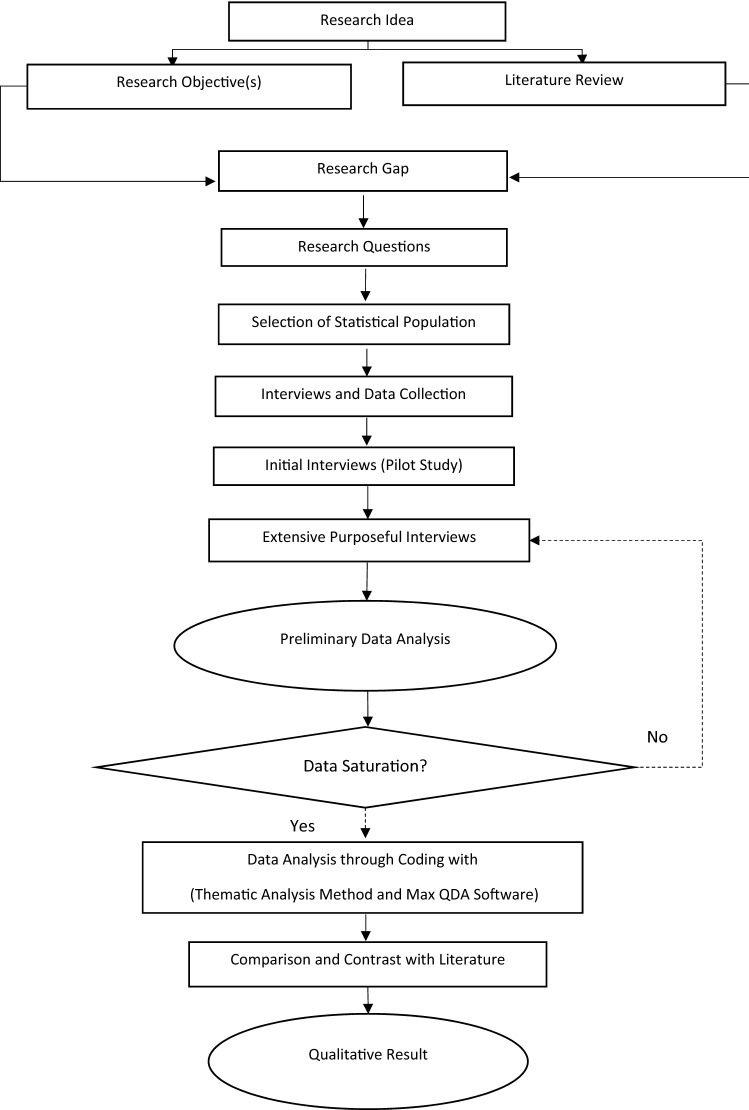
Research process flowchart of the study

Appropriate methodology for any study depends on the objectives and questions of the research. The literature review helps to formulate the research questions (Strauss and Corbin 1998) and establish a relevant methodology to accomplish the research. The aim of this study was to focus on a contemporary event (the Covid-19 pandemic) that does not require control over the phenomenon, and hence, qualitative research approaches are traditionally favored (Audet and d’Amboise 2001). Therefore, this study is a qualitative exploratory and explanatory type study that tries to gain an in-depth understanding of the business continuity challenges of the oil and gas sector. Process of the research is shown in Fig. 1.

### About the Pars Oil and Gas Company (POGC)

The Pars Oil and Gas Company (POGC) is one of the subsidiaries of the National Iranian Gas Company in the field of production and operation of onshore facilities of the South Pars Gas Company (SPGC) that is located in the territorial waters of Iran and Qatar in the Persian Gulf and is shared between the two countries. With an area of 9700 km^2^ and at a depth of 3000 m below the seabed level, it is the largest gas field in the world, about 105 km from the coast of Asaluyeh Port in Iran. The Iranian part of the field covers an area of 3700 km^2^ and its reserves are estimated at around 14 trillion m^3^ of gas and 17 billion barrels of gas condensate, equivalent to about 8% of the world’s gas reserves and 50% of Iran’s gas reserves.

The SPGC is located in Asaluyeh and Kangan Cities of Bushehr Province along the Persian Gulf coast in the form of two industrial complexes. Currently about 15,000 people work in the company. Most POGC employees commute to Asaluyeh, so there is a high risk of the employees spreading the virus to the larger communities. The POGC is responsible for the operation and refining of gas condensate and natural gas of the South Pars gas field. The company is Iran’s largest supplier of natural gas for domestic consumption and foreign export. The POGC owns and manages 10 gas refineries in the Pars Energy Special Economic Zone located in the Asaluyeh Port, Bushehr Province.^1^ The company plays a major role in the socioeconomic development and well-being of the region through employment and income generation and corporate social responsibility.

The POGC’s current goals are to meet the growing needs of the country for gas consumption, replacing oil with natural gas, increasing the share of gas in the domestic energy basket to save energy, and export gas, gas condensate, and liquefied petroleum gas (LPG). The vision of the POGC as the custodian of production from the South Pars gas field is the daily production of more than one billion m^3^ of natural gas and one million barrels of gas condensate (in total equivalent to 7.5 million barrels of crude oil). About two-thirds of the country’s gas is supplied from South Pars. In 2019, the natural gas production of the company reached about 700 million m^3^ per day, which had increased 2.5 times from the beginning of 2012. At this time 12 refineries are operating in the POGC. Gas exports also increased by 90% with the increase in South Pars gas production, and Iran now exports an average of about 80 million m^3^ of gas per day. Total gas production from South Pars gas field since the start of production in 1989 until March 2021 reached 1,867 billion m^3^ along with 2.2 billion barrels of gas condensates.^1^

### Data Collection

This study applied qualitative methods to understand the impacts of the Covid-19 pandemic on the POGC. Data were collected through field interviews (two field trips were taken) over a four-month period from March to June 2021. According to the objectives and research questions of the study, the purposive sampling method was adapted to collect the data through face-to-face, in-depth interviews.

For data collection, initially 15 senior executives were identified and after some screening, 8 key individuals were invited to participate in the study. We selected these individuals because: (1) they were the main operational managers of the POGC; (2) their departments were among those highly related to Covid-19 impacts and their management; and (3) they had the necessary information related to our research questions. Details of the interviewees (descriptive information) are presented in Table 1. All interviews were transcribed and saved in a text file. The study followed the research ethics protocols established by the Petroleum University of Technology (PUT) in Tehran.

**Table 1:** List of participants in the interviews

Interview no.	Position	Work experience at the company (years)	Work experience in the current position (years)	Age	Interview duration (minutes)
1	Managing Director	24	3	58	65
2	Executive Director	21	6	52	55
3	Head of Health, Safety, and Environment Department (HSE)	15	5	45	45
4	Head of Project Management Office (PMO)	16	6	44	50
5	Head of Procurement	18	6	51	63
6	Head of Logistics	17	5	53	60
7	Head of Quality Control	13	3	48	62
8	Head of Contracts and Finance	12	3	43	48

### Analytical Method

Content analysis generally involves the study and interpretation of texts and documents, some of which may be in the form of audio and visual materials that help to determine the extent of their spatial, temporal, and/or sequential occurrence. Thematic analysis is a process for analyzing qualitative information and is used in most qualitative studies. This method also allows the researcher to convert qualitative information into quantitative information (Buetow 2010; Gandasari and Dwidienawati 2020). The main difference between the two methods is that while content analysis focuses on transaction statistics and densities, thematic analysis extracts the intention of the text and the essence of the words. Thematic analysis, as a popular form of qualitative data analysis, helps researchers to identify emerging patterns from the set of events that one studies in content analysis. In this study, due to the emerging nature of the subject and the data related to Covid-19, thematic analysis was used.

To analyze the data using thematic analysis, a brief review of the interview texts was conducted to extract the main themes. A coding framework was developed based on the extracted themes. To code the text data features, an open coding approach (selecting topics to which the code is assigned and creating a list of topics and analytical notes) and axial coding approach (organizing the key concepts and reviewing and verifying the key codes) were used (Kulachinskaya et al. 2020). The codes were compared with the initial themes and were refined and screened iteratively. Subsequently, the theme network was drawn using the remaining codes from the previous stage. The emerged themes were ranked based on the number of repetitions in the text. The Max QDA 2020 software was used for coding and analysis of the data because of: (1) the ability to work with multiple data types; (2) the ability to store the project data in one project pack; (3) the ability to easily manage teamwork; (4) the quick and easy qualitative, statistical, and mixed methods analysis; and (5) the attractive visualizations (Kuckartz and Radiker 2019).

The validity and reliability of the findings of this study were assessed by careful considerations of the standard methods listed in the literature for qualitative analysis (Hammersley 1992; Morse et al. 2002). General familiarity with the POGC research site, allocating considerable length of time, building informal connections, and interactions with the POGC interviewees, helped the researchers gain an in-depth understanding of the POGC and its operations. In the data collection phase, interviews were recorded and transcribed and notes were taken. Important documents from different secondary sources (letters, instructions, statistics, orders, news, reports, and so on) were also collected and reviewed. For the data analysis, the use of thematic analysis methodology and its procedures was considered appropriate to ensure the validity and reliability of the data analysis. The host validation technique, which is suggested by Miles and Huberman (1994), was also used in this research. In doing so, a second field trip was conducted. The data were presented to the interview participants and the findings of the research were discussed and confirmed/refuted by them.

## Analysis and Findings

**Fig. 2:**
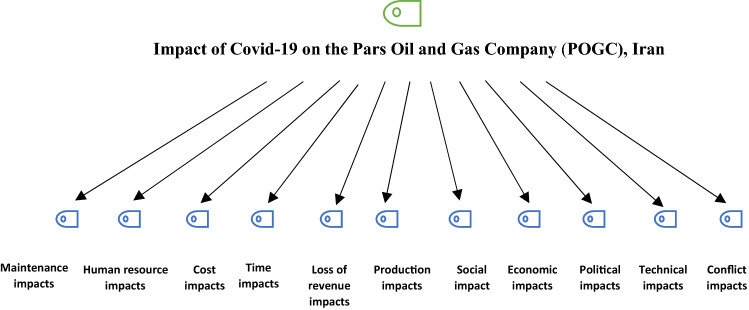
Hierarchical code (green) and sub-codes (blue) model

Figure 2 presents a summary of the key impact areas derived from the interviews using thematic analysis. According to these findings the impacts of the Covid-19 pandemic on POGC can be classified into 11 different operational, financial, and nonoperational categories, including maintenance, human resources, costs, delivery time, and revenue, as well as social, economic, political, and technical impacts.

In terms of the significance of the impacts, the maintenance process was found to have the highest score (19) (Table 2), that is, the interviewees emphasized the impact of the Covid-19 pandemic on the maintenance process 19 times. This emphasis on the maintenance operations can be explained by sub-codes and through mediating factors such as employee infections, staff mobility and transportation issues, and the impossibility of the timely presence of external pipelines inspectors. Human resources came in second with 18 points.

**Table 2:**
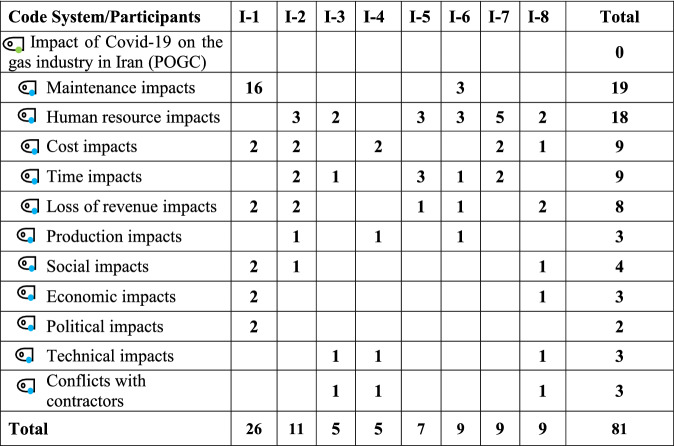
Cross-sectional table of scoring and dispersion of codes based on interviewee opinions (first phase)

*POGC* Pars Oil and Gas Company, *I* interviewee, *I-1* interviewee number 1

**Table 3:**
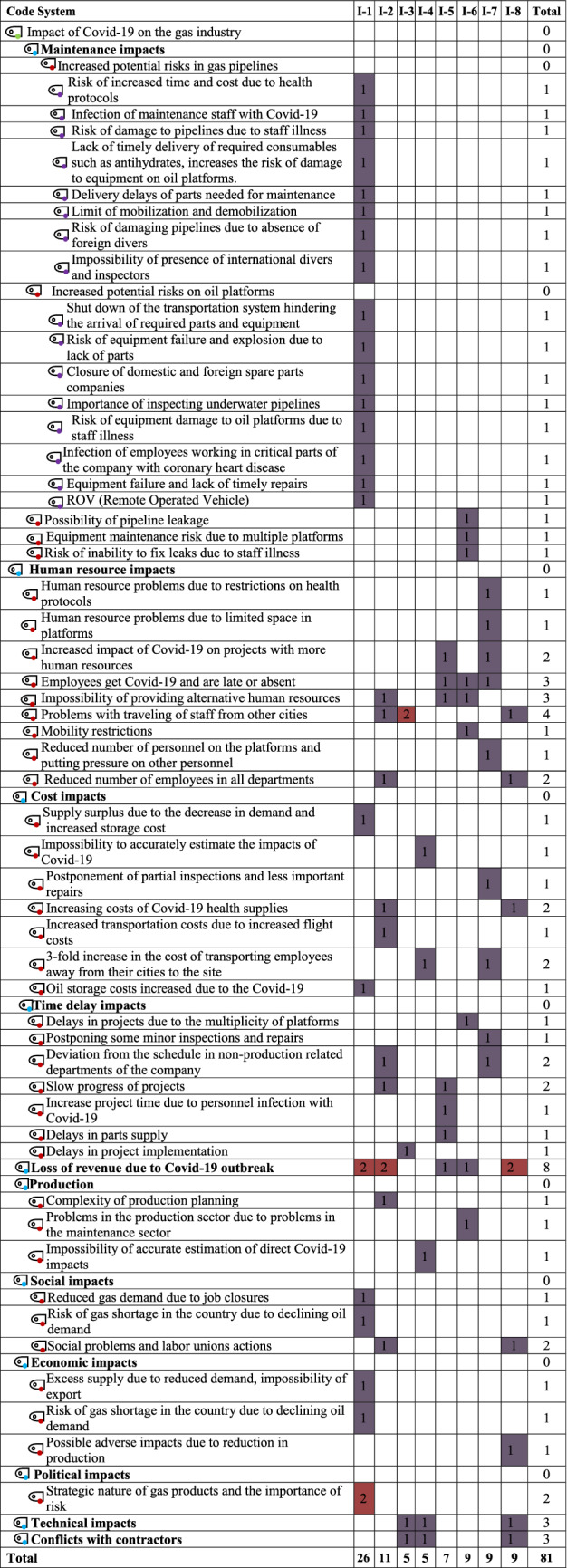
Cross-scoring table of codes based on interviewee opinions (second phase)

Table 2 shows the first phase and Table 3 shows the second phase of the research. In the first phase only the codes are displayed and in the second phase all the sub-codes are also displayed and the details are provided.

The interviewees from different departments and fields indicated that both operational and financial, as well as negative and positive, dimensions of Covid-19 on the POGC should be considered. Interviewee No. 8 (Table 1, in the field of Contracts and Finance) was very worried about the rising and additionally imposed costs: “Due to the delay in completing and running the ongoing projects, the loss of profits resulting from the Covid-19 crisis has reached more than 200 million dollars so far. The cost of personnel commuting to the site has increased three times due to the increase of flight ticket prices because airlines need to reduce the number of passengers to comply with Covid-19 protocols.” This interviewee also pointed out: “The exact number of damages and costs imposed on POGC projects after the outbreak of Covid-19 has not been accurately calculated so far. There should be some special rules and authorities. We need an independent committee to be formed to calculate these damages and make appropriate decisions.”

**Fig. 3:**
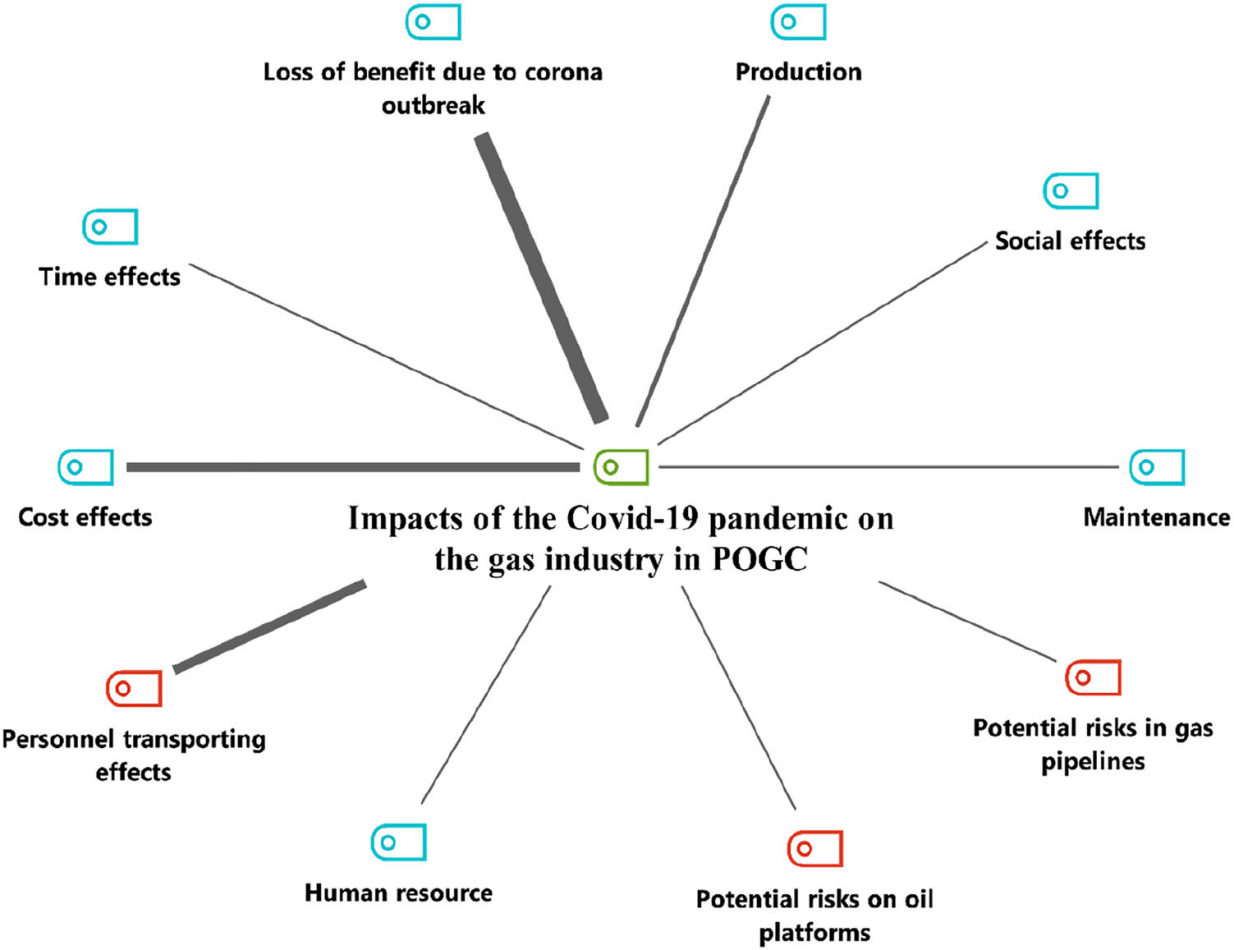
Multiple impacts of the Covid-19 pandemic on various aspects of the projects. POGC = Pars Oil and Gas Company

Interviewee No. 2 (Table 1) who is the Executive Director raised several issues: “The multifaceted and cascading impacts of Covid-19 on the POGC, such as financial impacts, conflicts with contractors, and labor problems. For example, quarantining infected personnel increases the costs, and conflict with contractors (due to the delays caused by the pandemic) has a significant impact on the financial sector.” Figure 3 shows the processes that were most important to the interviewees and that were mostly affected by the pandemic.

After conducting interviews with experts, in this study, 81 codes and sub-codes in total were recorded by the software and these codes were assigned to the content of the interviews based on the thematic analysis method. Participants’ codes are in columns and codes and sub-codes are in rows. For better identification and understanding, symbols with different colors are placed next to each code and sub-code. Green indicates the main title to which all codes and sub-codes are a subset. The blue color is placed after it and represents the main codes. Red indicates the main sub-codes and purple indicates the sub-codes. The main codes had the most repetition and emphasis among the interviewees and the sub-codes had the least emphasis by the interviewees. In Fig. 3, the thicker lines indicate that the interviewees placed more emphasis on these items, and the thinner lines indicate that fewer interviewees emphasized these items. But that does not mean they are not important and do not need to be examined and managed. All codes and sub-codes up to the third level along with their frequencies are presented in Table 3.

## Discussion

The findings of this study show that Covid-19 had multifaceted impacts on the POGC operations and their continuity. While the findings highlighted some of the key impacted areas and their possible sources, there are still many uncertainties about the actual impacts of the pandemic that need to be examined further. In particular, many uncertainties remain about the extent and exact values of the pandemic’s financial impacts. Similar interviews may be repeated to further understand the impacts over a longer period of time. This study was conducted during the third wave of the pandemic and the company has now passed the fifth wave. Over time the company has learned how to adapt to the situation and implement business continuity and risk management measures. Our findings show that the Covid-19 pandemic has had significant impacts on the POGC operations and continuity of business through 11 key areas. In some areas, the impacts are stronger than in others. Through the interviews a number of potential crisis management and business continuity measures were proposed and mentioned to reduce the impacts. For example, to minimize the impacts of Covid-19, regular meetings with contractors and employee representatives were suggested to minimize layoffs and labor actions. Furthermore, the contractors’ claims should be reviewed and processed on time and the scope of each contractor’s work should be re-examined carefully to identify the most vulnerable parts.

The Covid-19 pandemic has caused staff illness and as a result the cost of treating employees or buying health supplies for everyone to prevent the disease increased. Disease and quarantines also delayed production. Regarding employee health and safety, a number of measures were mentioned including the distribution of disinfectants and the availability of disinfection supplies for all employees, conducting training courses on Covid-19, periodic random tests, and the presence of a health team composed of doctors, nurses, and Health, Safety, and Environment (HSE) staff on all sites. Financial incentives and motivational systems such as rewards were also introduced and implemented to increase the morale of the staff during the pandemic.

To minimize the impact of transportation disruptions on the employees who commute to their workplace in the company, on-site accommodations were provided so that the employees could stay on site instead of commuting. To prevent further human and financial losses in the maintenance area, suggestions were made to review the existing technical inspection and periodic repair programs and adapt them to the pandemic situation. The company also decided to reprioritize its activities and operations and postpone those activities that have a lower criticality level during the pandemic.

Finally, it was important for the company to create a special Covid-19 crisis and risk management committee to guide and oversee the risk and continuity management policies and actions. The POGC needs new and agile ways of understanding and managing pandemics, to develop and implement new and more effective and objective rules, and to appreciate more coordination and communication to meet the rising expectations and overcome the crisis. The complexity and dynamics of Covid-19 were not completely perceived and dealt with among the managers and employees, although they have attempted to monitor, control, and minimize the negative impacts and, of course, in this crisis, they seek to turn threats into opportunities.

## Conclusion

This study revealed that the Covid-19 pandemic had significant impacts on POGC’s maintenance, human resources, processing and delivery times, operation costs, and revenues and caused some technical and contractual business continuity challenges for the company. However, maintenance and repair and production sectors were found to be the most impacted areas. These areas were impacted mostly by staff illness, lack of access to required parts and supplies, travel restrictions and cancellations, delays in equipment delivery, supply chain disruptions, and unavailability of international inspectors to inspect the underwater pipelines. Furthermore, this study revealed that the participants were concerned about the increasing risks to pipelines and platforms due to the inability to perform the required regular inspections, declining production, increasing technical and social issues, and increasing project times and costs. Many of these impacts were related to human resources that were directly and indirectly affected by the pandemic. Therefore, to minimize the impacts, special attention needs to be paid to human resources and employee health and safety, mobility, mental health, and the provision of appropriate welfare conditions to employees.

## Footnotes

1 https://www.pogc.ir/Default.aspx?tabid=136.
